# Surgical Techniques, Complications, and Long-Term Health Effects of Cardiac Implantable Electronic Devices

**DOI:** 10.7759/cureus.13001

**Published:** 2021-01-30

**Authors:** Crystal J Richardson, John Prempeh, Kyle S Gordon, Tracy-Ann Poyser, Frederick Tiesenga

**Affiliations:** 1 Internal Medicine, Saint James School of Medicine, The Quarter, AIA; 2 Internal Medicine, American University of Antigua, Osburn, ATG; 3 Internal Medicine, Windsor University School of Medicine, Cayon, KNA; 4 General Surgery, West Suburban Medical Center, Chicago, USA

**Keywords:** permanent pacemaker implantation (ppm), cardiac implantable electronic device (cied), cardiac resynchronization therapy defibrillator, implantable cardioverter-defibrillator, long-term care, postoperative complication

## Abstract

Cardiovascular implantable electronic device (CIED) has helped with advanced technological improvement in the cardiac field and has been a long-term alternative to medical management. There are different forms of CIEDs such as pacemakers, implantable cardioverter-defibrillators, and cardiac resynchronization therapy. These devices are efficient in establishing near-normal hemodynamics and circulation that ultimately aid physicians to improve the quality of life for their patients. However, there are risk factors that can result in postoperative complications, including infection, lead and pulse generator complications, heart complications, medication-related complications, and psychosocial complications. To ensure optimal outcome of CIED placement, preprocedural measures need to be in place such as matching the right candidate and using appropriate devices. This review aims to highlight the surgical techniques for CIEDs, the associated postoperative complications, and long-term health effects.

## Introduction and background

Over the last several decades we have seen technological advancements within the medical community and the cardiology field is no exception. As cardiac issues such as heart failure rise in America, leading to numerous challenges within the clinical setting, device therapy has been an excellent long-term alternative to normal medical management. Arrhythmias affect over 14.4 million Americans leading to over 400,000 deaths yearly [1].

Heart failure can predispose patients to various ventricular arrhythmias from ventricular tachycardia, ventricular fibrillation, or sudden cardiac death. Cardiac implantable electronic devices (CIEDs) are efficient in establishing near-normal hemodynamics and circulation [2]. Although medical management has evolved in the cardiology aspect, CIEDs have allowed physicians to enhance the quality of life for their patients and prevent sudden cardiac death.

There are different forms of CIED, however, the main three are pacemakers for bradyarrhythmias, implantable cardioverter-defibrillators (ICDs), and cardiac resynchronization therapy (CRT) [3]. CRT can further be divided into CRT defibrillators (CRT-D) or CRT pacers (CRT-P) [4]. While pacemakers are used to improve the quality of life for patients, CRTs are proven to improve ventricular ejection fraction, morbidity, and mortality in heart failure patients with reduced ejection fraction and left bundle branch block. It is also used to ease the uncomfortable symptoms accompanying heart failure patients [3,5]. Defibrillators tend to abort potential sudden cardiac death incidence and are used in patients related to abnormal ventricular activity.

Current peer-reviewed literature was studied to give a literature overview on the surgical techniques for CIED, its complications, and long-term health effects. Articles published in the English language, without geographic limitations, between the years of 1989 and 2020 were retrieved from databases including the American Heart Association, European Heart Journal, ScienceDirect, and PubMed. Only free articles were selected. The search excluded predatory publishers and predatory journals.

Studies that were included in the review were those on cardiac surgical techniques (such pacemakers, ICDs, and CRT). The search methodology was as follows: First, the word “cardiac implantable electronic devices” was searched for and the articles were narrowed down by going into the advanced search and typing AND “post-op complications,” “long-term health outcomes.” Second, articles were sorted out relevance. Third, the reference list of identified articles was analyzed to create a list of 33 articles that were included in the review.

## Review

Surgical techniques

A wide array of surgical techniques are utilized by surgeons when implanting CIEDs under various conditions whether under local or general anesthesia.

Pacemaker Implantation

For patients with cardiac issues such as sinus node dysfunction or tachyarrhythmias, to name a few, a permanent pacemaker implantation is indicated. Pacemakers detect abnormal cardiac impulses via wire-tipped electrodes and the computer generates electrical impulses transduced to the contracting myocardium to pace the heart [5].

Implantation of the pacemaker is normally performed in a cardiac catheterization or electrophysiology lab under local anesthesia. Patients are given antibiotic prophylaxis with first-generation cephalosporin cefazolin similar to other routine surgical procedures. A transvenous percutaneous approach is utilized to gain access to the cephalic vein, as well as to the left or right subclavian vein via the middle inner third of the clavicle and first rib.

After the subclavian vein is successfully punctured, a subcutaneous pocket is created where the pacemaker is implanted in the infraclavicular area. Subsequently, using fluoroscopy, the clinicians feed the pacemaker’s wire leads with a guide wire to enter the superior vena cava or the right atrium. The leads are successfully fed into the right atrium and sutured into the atrial wall utilizing non-absorbed sutures. Chest X-rays are taken in anterior-posterior and posterior-anterior positions to confirm the leads and the pacemaker implant are securely in place.

Subcutaneous Implantable Cardiac Defibrillators (S-ICDs)

S-ICDs are another form of CIED, the implantation technique used is primarily focused on creating pockets between muscle or fascia layers. Submuscular pocket implantations indicate for the defibrillator to be placed under the serratus anterior muscle (SAM). An incision is initially made in the inframammary crease using a “blunt dissection” technique, and the submuscular pocket is created for the CIED/S-ICD [2]. The surgeon locates the long thoracic nerve which innervates the SAM to preserve nerve and muscle function. Long-term complications such as winging of the scapula may ensue; however, this technique has been noted to reduce complications such as device erosion [1,2].

Subfascial pocket implantation calls for the CIED/S-ICD to be placed under the fascia using the same blunt dissection technique, as described above. The fascia is dissected from the SAM which is more superficial and less demanding than the subcutaneous methods which requires a more intrusive approach. The subfascial technique introduced an alternative to the subcutaneous method by allowing minimal postoperative pain, with the same cosmetic advantage as the prior technique [2]. Postsurgical clinical follow-ups indicate that there were no significant differences in appropriate or inappropriate shocks delivered among the different implantation (techniques) groups.

Cardiac Resynchronization Therapy

CRT is employed to restore automaticity and synchronize the atrial-intraventricular contractions in patients with severe CHF and widened QRS complex [3,4]. Implantation of the LV pacing leads is achieved by cannulating one of the coronary sinus tributaries via a transvenous approach [4]. The proximal coronary sinus is then intubated using a guiding sheath, with the pacing lead fed through one of the numerous branches of the coronary sinus to pace the lateral LV wall.

Researchers noted numerous concerns on the technical and, in particular, the anatomical aspects of the techniques used. The variations in venous anatomy among patients presented implantation complications of the left ventricular pacing lead. Difficulties that were noted included but are not limited to accessing the coronary sinus or the placement of the LV pacing lead in the lateral ventricular wall and voiding phrenic nerve stimulation [2,4].

We noticed in certain patients with technical difficulties or anatomical variations, different techniques were preferred. Surgical epicardial LV lead implantation was the ideal technique for optimal cardiac resynchronization, in particular, with patients who have transmural myocardial scarring [4].

Postoperative complications

Ideally, with the advancement of therapy (e.g., CIEDs), patients with comorbidities (e.g., cardiac disease) are expected to live longer. The increase in the likelihood of developing cardiovascular morbidities with increasing age is directly proportional to that of its therapeutic indication (e.g., CIED implantations) and associated complications. Although CIEDs are generally lifesaving, it is often associated with psychosocial effects, lead and pulse generator complications, heart malfunction (i.e., arrhythmias), and death. The most vulnerable group are pacemaker-dependent patients [6,7].

Most studies show a CIED-related complication risk of 5-6%. In a prospective study, a cohort of 114,484 aged 65 years or above with a median follow-up of 2.7 years showed a hospitalization or reoperation rate of 6.1%. Moreover, patients with postoperative complications onset within three months had the highest mortality rate at one and three years [8-10].

Infections

Mostly, CIED implantations are undertaken in an operating room under optimal sterile conditions. Infections have been associated with the skin microbiota (*Staphylococcus aureus*, *S. epidermidis*, and *Enterococci* species) and allergic reactions (recurrent infections). Suspicion of CIED infections usually results in device and blood culture, transesophageal echocardiogram, positron emission tomography, and computed tomography [11,12].

Infections that present acutely can be a life-threatening complication that occurs either intraoperatively or subsequently requiring the complete removal of CIEDs and administration of antibiotic therapy. However, a small subset of patients may choose to leave the CIED in place and receive palliative antibiotic treatment. The use of antibiotic pouches for prophylaxis could be useful in infection prevention. However, the efficacy is yet to be published [11-14].

Chronic infections, however, may have a late onset (e.g., six months postoperatively). Typically, these infections involve microbiota organisms (e.g., *S. epidermidis*) with low virulence and are associated with device erosion towards the superficial subcutaneous tissue, patients with minimal subcutaneous tissue, and those that underwent significant weight loss [11-13,15,16].

Lead and Pulse Generator Complications

Owing to the technological advances in CIEDs, device failures are typically detected by the device itself. Studies have shown that CIED-related mortality is extremely rare. Compared to older devices, novel devices offer both pacing and defibrillation. Classically, patients present with complaints of syncope and dizziness in the event of device failure. Patients who are primarily CIED-dependent have relatively worse presenting symptoms. Ironically, studies have shown more novel devices have a higher propensity to fail due to their complexity. Unfortunately, the contributing factors to these failures remain unidentified. In a prospective study, 2.9% out of a 1,317 cohort experienced lead malfunction in a 6.4-year span requiring revision. Lead failures were attributed to insulation defects (26%), artifact oversensing (24%), and lead fractures (24%). Recurrent CIED-related problem rates of 20% at five years have been reported [17-21].

Dislodgment and malfunction of the CIED pulse generator can occur in the event of malrotation in the pocket. The presenting symptoms usually include bradyarrhythmias (e.g., presyncope) due to lead restraints and higher bradycardia pacing threshold effects. In some cases, CIED malpositioning could be fatal due to failure in detecting and fixing arrhythmia. Precautionary measures aimed at appropriate suturing of the device to the pectoralis fascia in a fitting pocket and educating patients on the effects of mechanical pulling reduce the chances of malrotation [22,23].

Device hardware failures are an uncommon yet potential complication that could lead to death. Studies have shown the incidence of such an event ranging 0.01-0.1%. Intrinsic hardware failures (e.g., electronic circuit failure) and extrinsic hardware failures (e.g., cardioversion, radiation, and electromagnetic interference) are associated with hardware failures [24]. Current recommendations to minimize intrinsic and extrinsic failures include routine surveillance of device function and wearing protective gear when exposed to radiation (e.g., X-ray) [18,25,26].

Heart Complications

CIED-related arrhythmias are a potential complication often arising from inappropriate shocks. Paradoxically, CIEDs could trigger arrhythmias when attempting to attenuate supraventricular tachycardias (SVT) or any arrhythmia besides ventricular tachyarrhythmias. The most common SVT associated with inappropriate shocks is atrial fibrillation. Lead malfunction and enhanced sensing are other causes of inappropriate shocks. Studies have shown that novel dual chamber CIEDs and device programming tailored towards inappropriate shock prevention are paramount to reducing inappropriate shocks. Phantom shocks are another associated complication with CIED implantation. Phantom shocks should be suspected when device interrogation proves otherwise [27,28].

Damage to the endocardial architecture is a potential CIED-related complication sometimes fatal in the event of heart failure. Tricuspid regurgitation can result from the mechanical impedance of the leads or damage to the tricuspid valve. The incidence rate of developing is 10-20%, and about half of those patients end up with heart failure [29].

Psychosocial Complications

Appropriate shocks have been associated with psychosocial complications. Studies have shown about a 50% occurence of CIED-related psychosocial effects (e.g., anxiety and depression). Psychosocial complications are associated with anticipation of shock distress and lifestyle changes (e.g., changes in social life/physical activity and inability to drive or work). In a study of 119 CIED recipients, the majority tolerated the shocks (72%), some feared the shocks (23%), and some would rather be CIED-free (5%) [28,30].

Studies have shown that individual and group and cognitive and behavioral therapies have shown success in reducing the psychological effects of CIED-related complications. Measures to reduce the frequency of shocks in recurring cases should be undertaken to minimize the psychosocial burden the device has on patients [28,31-33].

## Conclusions

CIEDs have had rapid advancements in the last 70 years. Starting with a bulky external table top pacemaker that successfully provided treatment for heart block to the microprocessor-driven pacemakers that can monitor arrhythmias, bradycardic and tachycardic pacing, provide resynchronization for heart failure, and defibrillation.

All surgeries, especially CIED implantation, carry risks, including infection, lead and pulse generator complications, tricuspid regurgitation, arrhythmias, medication complications, and psychosocial complications. There are preprocedural measures that can be taken to ensure an optimal outcome in CIED placement. Most important is matching the right candidates and appropriate CIED device taking into consideration body habitus, preexisting health conditions, and comorbidities. Other considerations include experienced surgeon and medical staff, number of leads on CIED device, possible decolonization of *S. aureus* or *S. epidermidis* using chlorhexidine skin washing, and prophylactic antibiotic therapy. Following the operation, anticoagulation medications, appropriate wound management, early reintervention in case of lead displacement or suspected infection, and routine follow-up care are all factors to reduce adverse effects.

Cardiac muscle cells have an innate ability to conduct electrical impulses and begin beating even when a collection of cells are grown in a petri dish. However, when the heart is beating abnormally causing symptoms in the patient such as syncope or thromboemboli, CIEDs offer a chance for the patient to live a normal life.
